# Selection of GABA-Producing Lactic Acid Bacteria Strains by Polymerase Chain Reaction Using Novel *gad*B and *gad*C Multispecies Primers for the Development of New Functional Foods

**DOI:** 10.3390/ijms252413696

**Published:** 2024-12-21

**Authors:** Susana Langa, Silvia Santos, José Antonio Flores, Ángela Peirotén, Susana Rodríguez, José Antonio Curiel, José María Landete

**Affiliations:** Food Technology Department, National Institute for Agricultural and Food Research and Technology (INIA-CSIC), Carretera de La Coruña Km 7.5, 28040 Madrid, Spainangela.peiroten@inia.csic.es (Á.P.); susana.rodriguez@inia.csic.es (S.R.); joseantonio.curiel@inia.csic.es (J.A.C.); landete.josem@inia.csic.es (J.M.L.)

**Keywords:** gamma-aminobutyric acid, GABA, functional foods, health, molecular detection, PCR, gad, multispecies primers, bioactive compound, lactic acid bacteria

## Abstract

Gamma-aminobutyric acid (GABA) has been attributed to health-promoting properties and has received attention from the food industry as an attractive bioactive compound for the development of functional foods. Some lactic acid bacteria (LAB) produce GABA through a glutamate decarboxylase encoded by *gad*B and a glutamate/GABA antiporter encoded by *gad*C. In this study, we develop a molecular screening method based on a polymerase chain reaction able to detect those genes in different LAB species through the use of novel multispecies primers. PCR was performed in 92 LAB strains of six different species. The primer pair designed for *gad*B allowed its identification in *Lactiplantibacillus plantarum*, *Lactococcus cremoris*, *Lactococcus lactis*, *Levilactobacillus brevis*, *Limosilactobacillus fermentum*, and *Limosilactobacillus reuteri* strains. For *gad*C, two different primer pairs were designed for its detection in different species. Glutamate decarboxylase activity (GAD assay) and GABase enzymatic quantification were also assessed. Among those strains showing glutamate decarboxylase activity, 93.2% harbored the *gad*B gene, and those showing GABA production had the *gad*B gene and exhibited glutamate decarboxylase activity. PCR detection of *gad*B correlates strongly with GABA production and constitutes a good strategy for the selection of LAB with high yields (>18 mM) that could be used for the development of GABA-enriched functional foods.

## 1. Introduction

GABA is a major inhibitory neurotransmitter in the adult mammalian brain, considered a functional molecule with different situational functions in the central nervous system, the peripheral nervous system, and some non-neuronal tissues [1]. It has been known as an antihypertensive for decades when orally administered in supplements or fermented foods [2,3,4,5]. Moreover, it has other attributed physiological properties as an antidepressant [6] and an attributed cholesterol-lowering effect [7].

The ability to produce y-aminobutyric acid (GABA) through glutamate decarboxylase (GAD) constitutes an acid resistance mechanism found in some Gram-negative and Gram-positive bacteria [8,9,10,11]. The transformation of glutamate into GABA consumes an intracellular proton, increasing intracellular pH and providing protection in acidic conditions, which is an advantage for survival in the gastrointestinal tract or in fermented foods [12]. The production of GABA has been reported previously in LAB strains of a wide variety of species, such as *Lactococcus lactis*, *Lactiplantibacillus plantarum*, *Levilactobacillus brevis*, and many others [12,13,14,15,16].

GABA-producing LAB strains have been applied to obtain enriched fermented foods throughout fermentation [17]. GABA content in fermented foods depends on very different factors, such as the food matrix (dairy or vegetable), time of ripening, temperature, pH, starter cultures, or indigenous microbiota [18,19]. Thus, isolating LAB strains that are able to produce high GABA yields is an essential preliminary step for the development of GABA-enriched fermented foods.

In LAB, the GAD system, encoded by the *gad* operon, is responsible for glutamate decarboxylation and GABA secretion. The organization of this operon varies among different species and consists of two important elements: GAD encoded by either *gad*A or *gad*B and glutamate/GABA antiporter encoded by *gad*C [12,20]. Different authors have designed primers for detections of *gad*B and/or *gad*C in LAB [11,21,22,23], but these primers were species-specific. Therefore, our objective was to develop a molecular method for the detection of the genes involved in GABA production that could be applied to identify potential GABA-producing strains belonging to different LAB species. For this purpose, we developed non-degenerate and degenerate primers, which were designed by multiple sequence alignments of *gad*B and *gad*C genes from strains of different LAB species and genera for the detection of these genes by polymerase chain reaction (PCR). This molecular method will be more specific and will enable faster screening for GABA-producing strains belonging to different LAB species.

Hence, in this work, we tested the ability to produce GABA in 92 LAB strains belonging to the INIA bacterial collection by detecting *gad*B and *gad*C genes by PCR using the designed primes as well as determining their glutamate decarboxylase activity via a colorimetric method [8] and by the quantification of GABA by means of the GABA enzymatic assay.

## 2. Results

### 2.1. Optimization of PCR Conditions and Subsequent Detection of *gad*B and *gad*C Genes in LAB Strains

The alignments of the genes *gad*B or *gad*C of LAB strains presented in Table 1 did not show conserved regions suitable for designing non-degenerate primers for all of those species. Hence, degenerate primers for *gad*B detection were designed with the sequences of *gad*B from strains belonging to *Lactococcus* sp., *L. brevis*, *Limosilactobacillus fermentum*, and *L. plantarum*, while for the detection of *gad*C in LAB strains, we designed two pairs of primers. Therefore, F.gadC1/R.gadC1 primers were designed for *L. brevis* and *L. plantarum*, and degenerate primers F.gadC2/R.gadC2 were designed for *Lactococcus* sp. or *Limosilactobacillus reuteri* stains (Table 1).

Optimization of PCR conditions using the designed primers is shown in Table 2. The primer pair for *gad*B detection required a lower concentration than the other two (0.25 M), since higher primer concentrations (0.5 or 1 M) improved gene amplification, but some non-specific bands appeared in some of the LAB strains. The pair F.gadB-R.gadB amplified a 304-nucleotide fragment from *gad*B in all of the strains used for the optimization, i.e., *Lactococcus cremoris*, *L. lactis*, *L. brevis*, and *L. plantarum* strains (Table 2). The pair F.gadC1/R.gadC1 amplified a 397-nucleotide fragment from *gad*C in *L. brevis*, while the pair F.gadC2/R.gadC2 amplified a 184-nucleotide fragment from *gad*C in. *L. lactis* and *L. cremoris* strains (Table 2).

**Figure 1:**
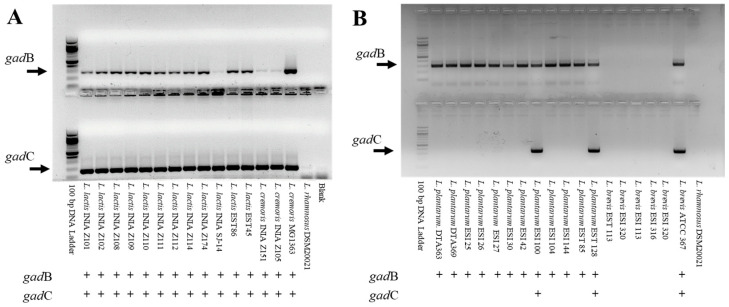
Representative gel electrophoresis of PCR products using primer pairs F.gadB/R.gadB for *gad*B detection in all strains tested; F.gadC2/R.gadC2 for *gad*C detection in *L. lactis* and *L. cremoris* (**A**); and F.gadC1/R.gadC1 for *gad*C detection in *L. plantarum* and *L. brevis* (**B**). The solid arrow indicates the predicted size of the amplicon. *L. cremoris* MG1363 and *L. brevis* ATCC367 were included as positive controls and *L. rhamnosus* DSM 20021 as negative control. (+) There is an amplification band and the gene is detected. (−) There is not amplification band and the gene is not detected.

We performed PCR using the designed primers and the optimized conditions in 92-LAB strains belonging to different species. PCR analysis revealed 63 positive strains for *gad*B, while only 29 were positive for both *gad*B and *gad*C (Table 3 and Table 4). The gene *gad*B was detected in *L. plantarum*, *L. fermentum*, *L. reuteri*, *L. lactis*, and *L. cremoris* strains. On the other hand, the primer pair F.gadC1/R.gadC2 showed amplification in only two *L. plantarum* strains, while the couple F.gadC2/RgadC2 resulted in positive results in 22 *L. lactis* and 5 *L. cremoris* strains (Figure 1).

Five out of the eight *L. cremoris* strains tested positive for *gad*B and *gad*C. Among the 42 strains tested, 27 harbored *gad*B, and 22 also had *gad*C. The gene *gad*B was detected in all except 2 of the 29 *L. plantarum* strains, but only 2 of them were positive for *gad*C. Among the *L. brevis* strains, none were positive for the presence of *gad*B or *gad*C. Three out of four *L. fermentum* strains and one out of four *L. reuteri* strains tested positive for *gad*B, while none of them were positive for *gad*C (Table 3 and Table 4).

### 2.2. Screening of GABA-Producing LAB Strains by the Glutamate Descarboxylase (GAD) Assay

Among the 92 LAB strains included in this study, 59 tested positive in the GAD assay as they showed color change after incubation in the GAD reagent (Table 3). Positive strains belonged to *L. plantarum* (28/29 strains), *L. cremoris* (1/8 strains), *L. lactis* (25/42 strains), *L. fermentum* (3/4 strains), and *L. reuteri* (2/4 strains). A total of 93.2% of the strains positive in the GAD assay harbored the *gad*B gene, while only 49.1% harbored *gad*C (Table 4). All *L. lactis* strains positive for the GAD assay were also positive for *gad*B, and most of them for *gad*C, while among the *L. cremoris* with *gad*B, only one showed GAD activity.

### 2.3. Quantification of GABA in Supernatants of LAB Strains by GABase

GABA production in LAB strains was detected and quantified by the GABase enzymatic method. For this, we used supernatants obtained from cultures incubated in MRS-MSG. Detectable amounts of GABA were recorded by GABase in 44 LAB strains (Table 3). All strains tested positive for the GAD assay and *gad*B gene and belonged to *L. plantarum* (22 strains), *L. lactis* (20 strains), and *L. fermentum* (2 strains). Most of these strains exhibited low amounts of GABA in supernatants. Nevertheless, significantly higher GABA yields were observed in several *L. lactis* strains (INIA Z108, INIA Z109, INIA Z110, INIA Z111, and INIA Z1174), while the highest amount of GABA was exhibited by the *L. plantarum* DTA 369 strain (Table 4).

## 3. Discussion

The isolation of LAB strains able to produce high GABA yields is of great interest to the food and pharmaceutical industry because of their potential as psychobiotics, defined as living microorganisms able to improve mental wellness by the release of neuroactive substances, such as GABA [24]. This inhibitory neurotransmitter is known to produce a calming effect as it plays a significant role in controlling nerve cell hyperactivity associated with mental health issues like anxiety, fear, and stress. Moreover, studies with germ-free mice have highlighted the effect of gut microbiota on GABA production and the mental health of individuals [25,26].

In this study, we have developed a PCR method based on multispecies primers to detect LAB strains harboring the genes implicated in GABA production and, hence, potential GABA producers. This molecular method will be more specific and will enable faster screening for GABA-producing strains belonging to different LAB species, as it only requires a PCR reaction from colony suspensions without requiring a previous DNA extraction protocol. This method has been tested with only 92 strains, and it is limited to 6 LAB species. A higher number of strains and species should be tested to evaluate this method in other LAB species, especially in the case of primers specifically designed for *gad*B.

A detection method for the *gad*B gene in GABA-producing strains using species-specific primers for *L. lactis*, *L. plantarum*, and *S. thermophilus* was previously developed by Valenzuela et al. [22]. On the other hand, Laroute et al. [21] designed specific primers for the detection of *gad*B and *gad*C in *L. lactis* and *L. cremoris*. In the present study, we aligned the *gad*B sequences from *L. brevis* ATCC376, *L. lactis* IL1403, *L. fermentum* F6, and *L. plantarum* KACC92189, not finding suitable consensus sequences for the development of primers that would allow the detection of these genes. Therefore, it was necessary to develop degenerate primers to overcome variations between sequences of different LAB species. The design of degenerate primers in this study for the detection of *gad*B in different LAB species and genera allowed us to detect this gene in strains belonging to *L. brevis*, *L. cremoris*, *L. fermentum*, *L. reuteri*, *L. lactis*, and *L. plantarum*.

The development of specific primers for *gad*C detection has been more complex as the alignment of *gad*C sequences of different LAB species showed the difficulty of developing a single primer pair that could work for the different LAB species mentioned above. Thus, in this case, we designed two different primer couples for *gad*C detection. Primer pair F.gadC1/R.gadC1 was designed for *gad*C detection in *L. brevis* and *L. plantarum* strains. The other pair of degenerate primers for *gad*C (F.gadC2/R.gadC2) was designed for lactococci and *L. reuteri* and worked with *L. cremoris* and *L. lactis*, but we could not detect *gad*C in any *L. reuteri* or *L. fermentum* strain tested.

From this analysis, we can conclude that *L. plantarum* DTA 369, with only *gad*B, exhibited the highest GABA yields of all strains tested, while other *L. plantarum* strains with both *gad*B and *gad*C showed significantly lower yields. In contrast, *L. lactis* INIA Z108, INIA Z109, INIA Z110, INIA Z111, and INIA Z174 strains, with the highest GABA yields (>18 mM), were positive for both genes and to the GAD assay, while strains with *gad*B, but not *gad*C, had significantly lower GABA production.

Results showed a good correlation between the detection of *gad*B and GAD activity. A total of 87.3% of the strains with the gene tested positive in the GAD assay, while GABA production was detected in 69.8% of the strains positive for *gad*B. Moreover, *L. lactis* INIA Z108, INIA Z109, INIA Z110, INIA Z111, and INIA Z174 strains, with the highest GABA yields (>18 mM) among the *L. lactis* species, were positive for *gad*B and *gad*C. In contrast, the only two *L. plantarum* strains harboring both *gad*B and *gad*C had significantly lower GABA production than the strain *L. plantarum* DTA 369, only positive for *gad*B, which exhibited the highest GABA yield of all of the strains tested (24.68 mM). This strain is interesting as it also has the ability to produce riboflavin. Moreover, riboflavin overproducing spontaneous mutants from this strain have been used to ferment soy beverages, showing not only the capacity to grow and ferment soy beverages but also to produce high yields of riboflavin during fermentation in a previous study [27].

Regarding *L. cremoris* strains, the lack of GABA production, in spite of the presence of the genes *gad*B and *gad*C in many of the strains tested, is in concordance with the finding of other authors, who describe that the gene *gad*B may be inactive in many *L. cremoris* strains, while strains belonging to *L. lactis* are better GABA producers [21,22,28].

Surprisingly, none of the *L. brevis* strains tested were positive for the genes of the production of GABA, even though this species has been described to have a high GABA-producing capability [29].

Typically, the *gad* operon is located on the chromosomes of LAB species, with its organization being highly variable among different species and strains [12]. In lactococci, there is a similar genetic organization of the GAD system [21,30] with a positive regulator encoded by *gad*R and expressed from the PgadR promoter and the *gad*CB operon consisting of *gad*C (encoding glutamate GABA antiporter) and *gad*B (encoding GAD) located downstream of the PgadCB promoter, ensuring that *gad*B and *gad*C genes are co-transcribed under the same promoter, similar to the GAD system described for *L. brevis* [31,32]. Regarding GABA-producing *L. plantarum*, some authors have already described the lack of a specific glutamate/GABA antiporter gene (*gad*C) next to *gad*B [11,32], which is consistent with the results obtained in this work, as only 2 out of the 22 GABA-producing strains were positive for this gene.

Based on these results, the PCR detection of *gad*B constitutes a good strategy for the selection of potential GABA-producing LAB strains, reducing the number of strains to be further analyzed for GABA quantification. Finding GABA-producing LAB strains is of great importance to the food industry as most of their species are recognized as safe, hold the QPS status [33], and are able to ferment different food matrixes. In this study, we have selected up to five high GABA-producing strains, four *L. lactis*, and one *L. plantarum*, which will be used in further studies for the development of functional foods, such as GABA-enriched fermented vegetable beverages, as previously developed with other bioactive compounds by our group [27]. On the other hand, the optimization of fermentation conditions, such as temperature, pH, or glutamate addition, and/or the heterologous expression of *gad*B and/or *gad*C will be of great interest for obtaining higher GABA yields in these functional beverages.

## 4. Materials and Methods

### 4.1. Bacterial Strains

LAB strains used in this work were obtained from the bacterial collection of the National Institute for Agricultural and Food Research and Technology (INIA-CSIC). Ninety-two strains were selected belonging to the species *L. brevis*, *L. cremoris*, *L. lactis*, *L. fermentum*, *L. reuteri*, and *L. plantarum* (Table 3). *L. cremoris* MG1363, *L. lactis* IL1403, and *L. brevis* ATCC367, with *gad*B and *gad*C genes, and *L. plantarum* WCFS1, with *gad*B, were included as positive controls. *L. rhamnosus* DSM 20021, without *gad*B and *gad*C genes, was used as a negative control. The strains were maintained at 30 °C with 5% glycerol and grown in MRS (de Man, Rogosa and Sharpe) at 37 °C under anaerobic conditions, with the exception of *Lactococcus* sp. strains, which were grown in M17 broth supplemented with 0.5% glucose (GM17) for 24 h at 30 °C under aerobic conditions. All media were purchased from BD Biosciences (Le Pont de Claix, France).

### 4.2. Detection of gadB and gadC Genes in LAB Strains by PCR Using Multispecies Primer

The sequences of the genes *gad*B and *gad*C of LAB strains were retrieved from the NCBI GenBank database. Multiple sequence alignments of these sequences with Clone Manager Suite 7 (Sci Ed Central, Cary, NC, USA) were used to design the primers. Three pairs of primers were designed (Table 2), two of them being degenerated following UPAC nomenclature: R (AG), Y (C/T), E (T/G).

For DNA preparation, 2–3 colonies growing on solid media were resuspended in 50 µL of sterile water to be directly added to the PCR mix. Initial PCR conditions tested for *gad* genes detection were as follows: initial denaturation at 95 °C for 5 min; 35 cycles of denaturation at 94 °C for 20 s., annealing at 48 °C for 30 s and elongation at 72 °C for 30 s; and final elongation at 72 °C for 7 min, using DNA AmpliTools Master Mix (Bigtools B&M Labs., Madrid, Spain) and following the manufacturer’s instructions. In the case of the degenerate primers for the detection of *gad*B and *gad*C, different concentrations of primers (0.25–1.0 µM) and annealing temperatures (40–50 °C) were tested. PCRs were performed in a 2720 Thermal Cycler (Applied Biosystems, Foster City, CA, USA), and products were visualized using a 2% agarose gel in 0.5 X TAE (all from Sigma-Aldrich, St. Louis, MO, USA) with gel Red^TM^ (EMD Millipore, Burlington, MA, USA). Samples and a 100 bp DNA ladder were loaded using a dye purple loading buffer (New England Biolabs, Ipswich, MA, EEUU). After running at 130 V for 30 min, gels were visualized using Gel Do^TM^ XR+ (Bio-Rad, Hercules, CA, USA).

Once the PCR conditions were set up, we tested the presence of *gad*B and *gad*C genes with the designed primers in the 92 LAB strains. *L. cremoris* MG1363 and *L. brevis* ATCC367, and *L. rhamnosus* DSM 20021, were included as positive and negative controls, respectively.

### 4.3. Determination of the Glutamate Decarboxylase Activity (GAD Assay)

The 92 LAB strains (Table 1) were tested for glutamate decarboxylase activity by the colorimetric method described by Cotter et al. [8] and adapted to lactococci by Lacroix et al. [34]. Briefly, 5 mL of overnight cultures were centrifuged (5000× *g*, 20 min, 25 °C) and washed twice with 5 mL of 0.9% (*w*/*v*) NaCl solution. The resulting pellets were resuspended in 0.5 mL of the GAD reagent solution (1 g of L-glutamic acid, 0.3 mL/L of Triton X-100, 90 g/L of NaCl, and 0.05 g/L of bromocresol green, pH 4). All reagents used were purchased from Merck (Barcelona, Spain). After 4 h of incubation at 37 °C under anaerobic conditions, samples were examined for color change to green or blue, indicating GAD activity. Three independent experiments were carried out for each strain tested.

### 4.4. Determination and Quantification of GABA Production by the GABase Enzymatic Method

LAB strains were grown in their corresponding media for 24 h and then in MRS supplemented with 3.5% sodium glutamate (Merck) (MRS-MSG) under their optimal conditions for 48 h. Then, supernatants were obtained by centrifugation at 12,000× *g*, filter sterilized (0.22 µM), and frozen at −20 °C until further analysis. The quantification of GABA in supernatants was performed using a GABase microtiter plate assay as described by Tsukatani et al. [35] with some modifications. A GABase reagent was prepared in Tris-HCI buffer (80 mM, pH 9.0) containing 750 mL sodium sulfate, 10 mM dithiothreitol, 1.4 mM NADP+, 20 mM α-ketoglutarate, and GABase (0.07 U/mL). All reagents used were purchased from Merck. A total volume of 90 µL of the GABase reagent and 10 µL of a standard or sample solution, both diluted 1:2 in water, were added to each well of a 96-well microtiter plate (Thermo Fisher Scientific, Waltham, MA, USA). Blank wells without enzymes were included for each sample or standard. After 120 min of incubation at 30 °C, NADPH formation was measured at 340 nm using a microplate reader (Varioskan Flash, Thermo Electron Corporation, Helsinki, Finland). Data obtained (n = 6) were analyzed by ANOVA using a general linear model (SPSS Statistics 22.0. IBM Corp., New York, NY, USA). Comparison of means between different GABA-producing strains was carried out by Tukey’s multiple range test at *p* < 0.01.

## 5. Conclusions

In this study, we have developed different multispecies primers for the PCR detection of *gad*B and *gad*C genes in LAB, involved in the production and transport of GABA, respectively. By performing PCR using the designed primers for *gad*B, we were able to detect this gene within a pool of 92 strains belonging to six different LAB species. Similarly, the two primer pairs designed for *gad*C allowed the detection of this gene by PCR in four different LAB species. Based on these results, the PCR detection of *gad*B constitutes a good strategy for the selection of GABA-producing LAB strains, reducing the number of strains to be further analyzed for GABA quantification. Selected strains could be used for the development of GABA-enriched functional foods.

## Figures and Tables

**Table 1:** Multispecies primers designed for the amplification of *gad*B and *gad*C genes involved in the production of GABA.

Primers	Sequence (5′ -> 3′)	Primers
F.gadB	TGGGARAAGTTCTGTRYCTACTGG	*gad*B from *L. brevis* ATCC367, *L. lactis* IL1403, *L. fermentum* F6 and *L. plantarum* KACC92189
R.gadB	ACGTTCKYCARCCGGAAGTCCC	
F.gadC1	GCATTGTTCTACCAATGGATTCA	*gad*C from *L. brevis* ATCC367 and *L. plantarum* KACC92189
R.gadC1	TGTGGTGCCGACCCTTCGGCACC	
F.gadC2	GTTTCKTTYATTCTAGCTTATATGGG	*gad*C from *L. cremoris* MG1363 and *L. reuteri* AM LB1
R.gadC2	GCACTTAATGATARYTCTTTTTG	

**Table 2:** Results of PCR optimization for the detection of *gad*B and *gad*C genes with multispecies primers.

Primer Pair	F.gadB/R.gadB	F.gadC1/R.gadC1	F.gadC2/R.gadC2
Gen detected	*gad*B	*gad*C	*gad*C
Amplicon size (bps)	304	397	184
Annealing temperature and time	48 °C/30 s	48 °C/30 s	40 °C/30 s
Primers concentration (μM)	0.25	0.50	0.50
*L. cremoris* MG1363	+	−	+
*L. brevis* ATCC 367	+	+	−
*L. lactis* IL1403	+	−	+
*L. plantarum* WCFS1	+	−	−
*L. rhamnosus* DSM 20021 (negative control)	−	−	−

(+) Detected or (−) non detected amplification band with those primers and conditions.

**Table 3:** Summary of GABA-producing LAB strains by detection of glutamate decarboxylase activity by GAD assay (GAD activity); detection of *gad*B and/or *gad*C genes by PCR with multispecies primers; and determination by GABase enzymatic method in supernatants from 48 h cultures grown in MRS-MSG.

Species	Strains Tested ^1^	Positive to *gad*B ^1^	Positive to *gad*C ^1^	Positive by GAD Assay ^1^	Positive by GABAse ^1^
*L. cremoris*	8	5	5	1	0
*L. lactis*	42	27	22	25	20
*L. brevis*	5	0	0	0	0
*L. fermentum*	4	3	0	3	2
*L. reuteri*	4	1	0	2	0
*L. plantarum*	29	27	2	28	22
Total	92	63	29	59	44

^1.^ Number of strains.

**Table 4:** Detection of *gad*B and/or *gad*C genes by PCR with multispecies primers; detection of glutamate decarboxylase activity by GAD assay (GAD activity); and determination of GABA production (mM) by GABase enzymatic method.

Strain	F.gadB/ R.gadB	F.gadC1/ R.gadC1	F.gadC2/ R.gadC2	GAD Activity	GABase
*Lactiplantibacillus plantarum* DTA 211	+	−		+	1.14 ± 0.19 ^a,b^
*Lactiplantibacillus plantarum* DTA 330	+	−		+	0.75 ± 0.40 ^a^
*Lactiplantibacillus plantarum* DTA 332	+	−		+	ND
*Lactiplantibacillus plantarum* DTA 336	+	−		+	ND
*Lactiplantibacillus plantarum* DTA 348	+	−		+	2.26 ± 0.73 ^a–d^
*Lactiplantibacillus plantarum* DTA 350	+	−		+	3.95 ± 0.09 ^a–f^
*Lactiplantibacillus plantarum* DTA 351	+	−		+	ND
*Lactiplantibacillus plantarum* DTA 352	+	−		+	ND
*Lactiplantibacillus plantarum* DTA 358	+	−		+	0.96 ± 0.25 ^a,b^
*Lactiplantibacillus plantarum* DTA 363	+	−		+	2.80 ± 0.47 ^a–d^
*Lactiplantibacillus plantarum* DTA 365	+	−		+	1.96 ± 0.40 ^a–c^
*Lactiplantibacillus plantarum* DTA 369	+	−		+	24.68 ± 1.39 ^m^
*Lactiplantibacillus plantarum* ESI 100	+	+		+	1.85 ± 0.46 ^a–c^
*Lactiplantibacillus plantarum* ESI 104	+	−		+	2.59 ± 0.36 ^a–d^
*Lactiplantibacillus plantarum* ESI 144	+	−		+	3.23 ± 0.17 ^a–f^
*Lactiplantibacillus plantarum* ESI 25	+	−		+	6.55 ± 0.75 ^f,g^
*Lactiplantibacillus plantarum* ESI 26	+	−		+	2.92 ± 0.21 ^a–d^
*Lactiplantibacillus plantarum* ESI 27	+	−		+	4.04 ± 1.35 ^a–f^
*Lactiplantibacillus plantarum* ESI 28	+	−		+	2.60 ± 0.29 ^a–d^
*Lactiplantibacillus plantarum* ESI 298	−	−		+	ND
*Lactiplantibacillus plantarum* ESI 30	+	−		+	2.13 ± 0.50 ^a–d^
*Lactiplantibacillus plantarum* ESI 305	−	−		+	ND
*Lactiplantibacillus plantarum* ESI 357	+	−		+	6.36 ± 0.72 ^e–f^
*Lactiplantibacillus plantarum* ESI 38	+	−		−	ND
*Lactiplantibacillus plantarum* ESI 42	+	−		+	2.26 ± 0.56 ^a–d^
*Lactiplantibacillus plantarum* EST 128	+	+		+	2.06 ± 1.46 ^a–d^
*Lactiplantibacillus plantarum* EST 214	+		−	+	1.74 ± 0.22 ^a–c^
*Lactiplantibacillus plantarum* EST 37	+	−		+	1.33 ± 0.32 ^a–c^
*Lactiplantibacillus plantarum* EST 85	+	−		+	4.56 ± 1.07 ^c–f^
*Lactococcus cremoris* ESI 277	−		−	−	ND
*Lactococcus cremoris* INIA 450	−		−	−	ND
*Lactococcus cremoris* INIA Z105	+		+	−	ND
*Lactococcus cremoris* INIA Z113	+		+	−	ND
*Lactococcus cremoris* INIA Z127	+		+	−	ND
*Lactococcus cremoris* INIA Z151	+		+	+	ND
*Lactococcus cremoris* INIA Z72	+		+	−	ND
*Lactococcus cremoris* TAB 24	−		−	−	ND
*Lactococcus lactis* ESI 153	−		−	−	ND
*Lactococcus lactis* ESI 240	+		+	+	4.29 ± 0.23 ^b–f^
*Lactococcus lactis* ESI 515	−		−	−	ND
*Lactococcus lactis* ESI 561	−		−	−	ND
*Lactococcus lactis* ESI 718	+		−	+	2.19 ± 0.10 ^a–d^
*Lactococcus lactis* EST 187	−		−	−	ND
*Lactococcus lactis* EST 221	−		−	−	ND
*Lactococcus lactis* EST 30	−		−	−	ND
*Lactococcus lactis* EST 32	−		−	−	ND
*Lactococcus lactis* EST 45	+		+	+	ND
*Lactococcus lactis* EST 5	−		−	−	ND
*Lactococcus lactis* EST 70	−		−	+	ND
*Lactococcus lactis* EST 72	−		−	−	ND
*Lactococcus lactis* EST 86	+		+	+	ND
*Lactococcus lactis* EST 89	+		+	+	ND
*Lactococcus lactis* EST 91	+		+	+	ND
*Lactococcus lactis* INIA 12	−		−	−	ND
*Lactococcus lactis* INIA 13	−		−	−	ND
*Lactococcus lactis* INIA 14	−		−	−	ND
*Lactococcus lactis* INIA 415	+		−	+	3.03 ± 0.35 ^a–e^
*Lactococcus lactis* INIA 437	+		−	+	1.42 ± 0.14 ^a–c^
*Lactococcus lactis* INIA SJ-14	+		+	+	12.38 ± 0.19 ^i,j^
*Lactococcus lactis* INIA SJ-43	+		−	+	10.75 ± 0.27 ^h,i^
*Lactococcus lactis* INIA Z101	+		+	+	8.06 ± 0.79 ^g,h^
*Lactococcus lactis* INIA Z102	+		+	+	6.44 ± 0.31 ^e–f^
*Lactococcus lactis* INIA Z103	+		+	+	5.37 ± 0.18 ^d–f^
*Lactococcus lactis* INIA Z108	+		+	+	21.11 ± 0.90 ^l^
*Lactococcus lactis* INIA Z109	+		+	+	20.47 ± 2.04 ^l^
*Lactococcus lactis* INIA Z11	+		+	+	1.01 ± 0.17 ^a,b^
*Lactococcus lactis* INIA Z110	+		+	+	18.22 ± 3.82 ^k,l^
*Lactococcus lactis* INIA Z111	+		+	+	18.23 ± 2.15 ^k,l^
*Lactococcus lactis* INIA Z112	+		+	+	11.24 ± 0.37 ^h,i^
*Lactococcus lactis* INIA Z114	+		+	+	14.85 ± 0.91 ^j,k^
*Lactococcus lactis* INIA Z174	+		+	+	18.41 ± 2.67 ^l^
*Lactococcus lactis* INIA Z29	+		+	−	ND
*Lactococcus lactis* INIA Z34	+		+	−	ND
*Lactococcus lactis* INIA Z39	+		+	−	ND
*Lactococcus lactis* INIA Z53	+		+	+	2.06 ± 0.20 ^a–d^
*Lactococcus lactis* INIA15	+		+	+	1.22 ± 1.12 ^a–c^
*Lactococcus lactis* TAB 26	+		−	+	0.73 ± 0.50 ^a^
*Lactococcus lactis* TAB 50	−		−	−	ND
*Lactococcus lactis* TAB 75	−		−	−	ND
*Levilactobacillus brevis* ESI 113	−	−		−	ND
*Levilactobacillus brevis* ESI 316	−	−		−	ND
*Levilactobacillus brevis* ESI 317	−	−		−	ND
*Levilactobacillus brevis* ESI 320	−	−		−	ND
*Levilactobacillus brevis* EST 113	−	−		−	ND
*Limosilactobacillus fermentum* 584L	+		−	+	2.26 ± 0.19 ^a–d^
*Limosilactobacillus fermentum* 832L	+		−	+	ND
*Limosilactobacillus fermentum* INIA P143	+		−	+	3.71 ± 0.11 ^a–f^
*Limosilactobacillus fermentum* S900	−		−	−	ND
*Limosilactobacillus reuteri* INIA P570	−		−	−	ND
*Limosilactobacillus reuteri* INIA P572	−		−	−	ND
*Limosilactobacillus reuteri* INIA P576	−		−	+	ND
*Limosilactobacillus reuteri* INIA P580	+		−	+	ND

(+) Considered positive for *gad*B or *gad*C detection by PCR using those primers or positive for GAD activity. (−) Negative for *gad* genes or GAD activity. Values obtained by GABAse method are mean ± SD (n = 6). Superscripts (a–m) indicate significant differences according to Tukey’s test (*p* < 0.01). ND: Non detected.

## Data Availability

The data presented in this study are available on request from the corresponding author.

## References

[1] Watanabe M., Maemura K., Kanbara K., Tamayama T., Hayasaki H. (2002). GABA and GABA receptors in the central nervous system and other organs. Int. Rev. Cytol..

[2] Inoue K., Shirai T., Ochiai H., Kasao M., Hayakawa K., Kimura M., Sansawa H. (2003). Blood-pressure-lowering effect of a novel fermented milk containing gamma-aminobutyric acid (GABA) in mild hypertensives. Eur. J. Clin. Nutr..

[3] Liu C.F., Tung Y.T., Wu C.L., Lee B.H., Hsu W.H., Pan T.M. (2011). Antihypertensive effects of *Lactobacillus* fermented milk orally administered to spontaneously hypertensive rats. J. Agric. Food Chem..

[4] Matsubara F., Ueno H., Tadano K., Suyama T., Imaizumi K., Suzuki T., Magata K., Kikuchi N., Muneyuki K., Nakamichi N. (2002). Effects of GABA supplementation on blood pressure and safety in adults with mild hypertension. Jpn. J. Pharmacol. Ther..

[5] Watanabe T., Yamada T., Tanaka H., Hang S., Mazumder T.K., Nagai S., Tsuji K. (2002). Antihypertensive effect of γ-aminobutyric acid-enriched on spontaneously hypertensive rats. J. Jpn. Soc. Food Sci..

[6] Wu S.-J., Chang C.Y., Lai Y.T., Shyu Y.T. (2020). Increasing γ-aminobutyric acid content in vegetable soybeans via high-pressure processing and efficacy of their antidepressant like activity in mice. Food.

[7] Watanabe S., Katsube T., Sonomoto K. (2012). Cholesterol-lowering effects of isolated from Turnip “Tsuda Kabu”. Food Sci. Technol. Res..

[8] Cotter P.D., Gahan C.G., Hill C.A. (2001). glutamate decarboxylase system protects *Listeria monocytogenes* in gastric fluid. Mol. Microbiol..

[9] Cotter P.D., Hill C. (2003). Surviving the acid test. Responses of Gram-positive bacteria to low pH. Microbiol. Mol. Biol. Rev..

[10] Foster L.W. (2004). *Escherichia coli* acid resistance tales of an amateur acidophile. Nat. Rev. Microbiol..

[11] Yunes R.A., Poluektova E.U., Dyachkova M.S., Klimina K.M., Kovtun A.S., Averina O.V., Orlova V.S., Danilenko V.N. (2016). GABA production and structure of *gad*B gad genes in *Lactobacillus* and *Bifidobacterium* strains from human microbiota. Anaerobe.

[12] Yogeswara B.A., Maneerat S., Haltrich D. (2020). Glutamate decarboxylase from lactic acid bacteria a key enzyme in GABA synthesis. Microorganisms.

[13] Bhanwar S., Singh A., Ganguli A. (2013). Probiotic characterization of potential hydrolases producing *Lactococcus lactis* subsp. *lactis* isolated from pickled yani. Dt. J. Food Sci. Nutr..

[14] Edalatian Dovom M.R., Habibi Najafi M.B., Rahnama Vosough P., Norouzi N., Ebadi Nezhad S.J., Mayo B. (2023). Screening of lactic acid bacteria strains isolated from Iranian traditional dairy products for GABA production and optimization by response surface methodology. Sci. Rep..

[15] Komatsuzaki N., Shima J., Kawamoto S., Momose H., Kimura T. (2005). Production of γ-aminobutyric acid (GABA) by *Lactobacillus paracasei* isolated from traditional fermented foods. Food Microbiol..

[16] Redruello B., Saidi Y., Sampedro L., Ladero V., del Rio B., Alvarez M.A. (2021). GABA producing *Lactococcus lactis* strains isolated from camel’s milk as starters for the production of GABA-enriched cheese. Food.

[17] Lee X.Y., Tan J.S., Cheng L.H. (2023). Gamma aminobutyric acid (GABA) enrichment in plant-based food- a mini review. Food Rev. Int..

[18] Rayavarapu B., Tallapragada P., MS U. (2021). Optimization and comparison of y aminobutyric acid (GABA) production by LAB in soymilk using RSM and ANN models. Beni-Suef Univ. J. Basic Appl. Sci..

[19] Xiao T., Shah N.P. (2021). Lactic acid produced by *Streptococcus thermophilus* activated glutamate decarboxylase (GadA) in *Lactobacillus brevis* NPS-QW 145 to improve γ-amino butyric acid production during soymilk fermentation. LWT.

[20] Cui Y., Miao K., Niyaphorn S., Qu X. (2020). Production of gamma-aminobutyric acid from lactic acid bacteria: A systematic review. Int. J. Mol. Sci..

[21] Laroute V., Aubry N., Audonnet M., Mercier Bonin M., Daveran Mingot M.L., Cocaign Bousquet M. (2023). Natural diversity of lactococci in y-aminobutyric acid GABA production and genetic and phenotypic determinants. Microb. Cell Fact..

[22] Valenzuela J.A., Flórez A.B., Vázquez L., Vasek O.M., Mayo B. (2019). Production of γ-aminobutyric acid (GABA) by lactic acid bacteria strains isolated from traditional, starter-free dairy products made of raw milk. Benef. Microbes.

[23] Wu Q., Law Y.S., Shah N. (2015). Dairy Streptococcus thermophilus improves cell viability of Lactobacillus brevis NPS-QW-145 and and its γ-aminobutyric acid biosynthesis ability in milk. Sci. Rep..

[24] Dinan T.G., Stanton C., Cryan J.F. (2013). Psychobiotics: A Novel Class of Psychotropic. Biol. Psychiatry.

[25] Dhyani P., Goyal C., Dhull S.B., Chauhan A.K., Singh Saharan B., Harshita, Duhan J.S., Goksen G. (2024). Psychobiotics for Mitigation of Neuro-Degenerative Diseases: Recent Advancements. Mol. Nutr. Food Res..

[26] Matsumoto M., Kibe R., Ooga T., Aiba Y., Sawaki E., Koga Y., Benno Y. (2013). Cerebral low-molecular metabolites influenced by intestinal microbiota: A pilot study. Front. Syst. Neurosci..

[27] Langa S., Peirotén A., Rodríguez S., Calzada J., Prieto-Paredes R., Curiel J.A., Landete J.M. (2024). Riboflavin bio-enrichment of soy beverage by selected roseoflavin-resistant and engineered lactic acid bacteria. Int. J. Food Microbiol..

[28] Nomura M., Kobayashi M., Ohmomo S., Okamoto T. (2000). Inactivation of the glutamate decarboxylase gene in *Lactococcus lactis* subsp. *cremoris*. Appl. Environ. Microbiol..

[29] Sezgin E., Tekin B. (2023). Molecular evolution and population genetics of glutamate decarboxylase acid resistance pathway in lactic acid bacteria. Front. Genet..

[30] Sanders J.W., Leenhouts K., Burghoorn J., Brands J.R., Venema G., Kok J. (1998). A chloride-inducible acid resistance mechanism in *Lactococcus lactis* and its regulation. Mol. Microbiol..

[31] Gong L., Ren C., Xu Y. (2020). GlnR negatively regulates glutamate-dependent acid resistance in *Lactobacillus brevis*. Appl. Environ. Microbiol..

[32] Wu Q., Tun H.M., Law Y.S., Khafipour E., Shah N.P. (2017). Common distribution of *gad* operon in *Lactobacillus brevis* and its GadA contributes to efficient GABA synthesis toward cytosolic near-neutral pH. Front. Microbiol..

[33] Wessels S., Axelsson L., Hansen E.B., De Vuyst L., Laulund S., Lähteenmäki L., Lindgren S., Mollet B., Salminen S., von Wright A. (2004). The lactic acid bacteria, the food chain, and their regulation. Trends Food Sci. Technol..

[34] Lacroix N., St-Gelais D., Champagne C., Vuillemard J. (2013). Gamma-aminobutyric acid-producing abilities of lactococcal strains isolated from old-style cheese starters. Dairy Sci. Technol..

[35] Tsukatani T., Higuchi T., Matsumoto K. (2005). Enzyme-based microtiter plate assay for γ-aminobutyric acid: Application to the screening of γ-aminobutyric acid-producing lactic acid bacteria. Anal. Chim. Acta.

